# Osteonecrosis of the Femoral Head in Patients with Hypercoagulability—From Pathophysiology to Therapeutic Implications

**DOI:** 10.3390/ijms22136801

**Published:** 2021-06-24

**Authors:** Elena Rezus, Bogdan Ionel Tamba, Minerva Codruta Badescu, Diana Popescu, Ioana Bratoiu, Ciprian Rezus

**Affiliations:** 1 Department of Rheumatology and Physiotherapy, “Grigore T. Popa” University of Medicine and Pharmacy Iași, 16 University Street, 700115 Iasi, Romania; elena_rezus@yahoo.com (E.R.); ioanaharton@yahoo.com (I.B.); 2 I Rheumatology Clinic, Clinical Rehabilitation Hospital, 14 Pantelimon Halipa Street, 700661 Iasi, Romania; 3 Advanced Center for Research and Development in Experimental Medicine (CEMEX), “Grigore T. Popa” University of Medicine and Pharmacy Iasi, 9-13 Mihail Kogălniceanu Street, 700454 Iasi, Romania; bogdan.tamba@umfiasi.ro; 4 Department of Internal Medicine, “Grigore T. Popa” University of Medicine and Pharmacy, 16 University Street, 700115 Iasi, Romania; dr.popescu.diana@gmail.com (D.P.); ciprianrezus@yahoo.com (C.R.); 5 III Internal Medicine Clinic, “St. Spiridon” County Emergency Clinical Hospital, 1 Independence Boulevard, 700111 Iasi, Romania

**Keywords:** osteonecrosis, femoral head, hereditary thrombophilia, hypofibrinolysis, anticoagulant

## Abstract

Osteonecrosis of the femoral head (ONFH) is a debilitating disease with major social and economic impacts. It frequently affects relatively young adults and has a predilection for rapid progression to femoral head collapse and end-stage hip arthritis. If not diagnosed and treated properly in the early stages, ONFH has devastating consequences and leads to mandatory total hip arthroplasty. The pathophysiology of non-traumatic ONFH is very complex and not fully understood. While multiple risk factors have been associated with secondary ONFH, there are still many cases in which a clear etiology cannot be established. Recognition of the prothrombotic state as part of the etiopathogeny of primary ONFH provides an opportunity for early medical intervention, with implications for both prophylaxis and therapy aimed at slowing or stopping the progression of the disease. Hereditary thrombophilia and hypofibrinolysis are associated with thrombotic occlusion of bone vessels. Anticoagulant treatment can change the natural course of the disease and improve patients’ quality of life. The present work focused on highlighting the association between hereditary thrombophilia/hypofibrinolysis states and ONFH, emphasizing the importance of identifying this condition. We have also provided strong arguments to support the efficiency and safety of anticoagulant treatment in the early stages of the disease, encouraging etiological diagnosis and prompt therapeutic intervention. In the era of direct oral anticoagulants, new therapeutic options have become available, enabling better long-term compliance.

## 1. Introduction

Osteonecrosis, avascular necrosis or aseptic necrosis of bone are all defined as bone cell death following the compromise of blood flow to the bone [1]. The basic underlying mechanism is the cessation of circulation to a specific area [2]; both arteries and veins may be involved. The trauma leads to vascular interruption of arterial blood flow, which is mainly and directly responsible for bone ischemia. Glueck et al. [3] and Orth et al. [4] hypothesized that the intravascular coagulation which occurs in the thrombophilia-hypofibrinolysis states determines venous thrombotic occlusion—which leads initially to increased intraosseous venous pressure and subsequently to impaired arterial blood flow and bone ischemia. Regardless of the initiating mechanism, the interruption of blood flow leads to persistent osseous hypoxia and lack of nutrients, factors which are responsible for bone necrosis. The osteocytes die, leading to bone resorption and collapse of the articular surface.

Although it can occur in any bone, the most commonly affected site is the femoral head [5]. Osteonecrosis of the femoral head (ONFH) encompasses traumatic and nontraumatic causes, the first being by far the more prevalent [5]. The femoral head blood supply lies mainly on retinacular arteries and, to a lesser extent not exceeding 20%, on the artery of the ligamentum teres. Retinacular arteries (superior, inferior, anterior, posterior), originating from medial and lateral circumflex femoral arteries, wrap around the femoral neck on their way to the femoral head. Ligamentum teres is an intra-articular ligament between the femoral head and acetabulum. Traumatic lesions such as displaced femoral neck fractures and hip dislocations severely compromise the vascularity around the femoral head, leading to bone ischemia and ONFH [6].

Nontraumatic ONFH has traditionally been classified as idiopathic or secondary, depending on the absence or presence of known causes [5]. The pathophysiology of the nontraumatic ONFH is very complex, multifactorial and not fully known. Multiple risk factors have been associated with secondary ONFH. More than 80% of cases are a consequence of long-term corticosteroid use and excessive alcohol intake [5]. Hemoglobinopathies (such as thalassemia [7] and sickle cell anemia [8]) and coagulopathies, such as hemophilia [9] and congenital afibrinogenemia [10] are also associated with ONFH. The blood flow in the bone microcirculation is compromised by the rigid and less-deformable red blood cells in thalassemic patients [7] or by abnormally shaped and stiff red blood cells in patients with sickle cell anemia [8]. Blockages in microcirculation result in bone infarction. Acute hemarthrosis is associated with increased intra-articular pressure and impaired blood flow to the bone. Recurrent hemorrhages in the hip joint can lead to femoral head ischemia and ONFH in patients with congenital bleeding disorders [9,10]. Malignancies—including myeloproliferative disorders, autoimmune diseases (e.g., systemic lupus erythematosus, rheumatoid arthritis), metabolic disorders (e.g., diabetes mellitus, hyperlipidemia, lipid storage diseases) and renal failure have all been shown to be involved in ONFH [5,11,12,13,14]. Currently, a wide range of modern antineoplastic medications are linked to bone necrosis, including tyrosine kinase inhibitors [15,16,17], monoclonal antibodies [17,18], mammalian target of rapamycin inhibitors, radiopharmaceuticals, selective estrogen receptor modulators and immunosuppressants [17].

Still, in many studied cases, a clear etiology could not be established. ONFH was considered primary (idiopathic); however, it may be—at least in part—the consequence of hereditary thrombophilia or hypofibrinolysis.

In the early 1990s, it was suggested that the hypercoagulability state could be a risk factor for osteonecrosis [19]. Since then, much evidence has been gathered to support this hypothesis, including data from animal models [20,21,22] which confirmed that venous occlusion was a primary event. Recognition of the prothrombotic state as part of the etiopathogeny of primary ONFH provides an opportunity for early medical intervention, with implications for both prophylaxis and therapy aimed at slowing or stopping the progression of the disease. This is all the more important, as studies show that the highest incidence of nontraumatic ONFH is in relatively young adults (males aged 20 to 50 years) [6,23,24], and that hip joint destruction rapidly progresses into end-stage hip arthritis, requiring surgical intervention [25].

Since significantly high prevalence of coagulation abnormalities is reported in patients with primary ONFH, our review aimed to conduct an in-depth analysis of their implications in the occurrence and progression of this disease. Additionally, since patients with inherited thrombophilia and hypofibrinolysis benefit from anticoagulant treatment in prophylaxis and treatment of thrombotic events, we also focused on anticoagulant use in primary ONFH patients with a hereditary thrombophilia—hypofibrinolysis background.

## 2. Hereditary Thrombophilia Associated with ONFH

Familial thrombophilia includes a broad spectrum of genetic abnormalities that interfere with the coagulation cascade. Differences have been noted in their prevalence and capacity to determine thrombosis. The highest frequency of heritable thrombophilia was recorded in individuals with ancestry from northern Europe [26] and some parts of the Middle East [27]. The most common and important thrombophilic states are: the factor V Leiden mutation, the prothrombin gene G20210A mutation, antithrombin III deficiency, protein C and protein S deficiency [28] and the methylenetetrahydrofolate reductase (MTHFR) C677T gene polymorphism [29]. It has also been noted that thrombosis may occur due to either a single dominant abnormality or to a combination of milder but multiple defects—as by association, their prothrombotic effects are potentiated.

The modern view of hemostasis grants the platelets a very important role in thrombus formation [30]. In the cell-based coagulation model, platelet and coagulation factors are strongly interconnected, since platelets provide rich phosphatidylserine-exposing surfaces on which high levels of thrombin are produced and, in turn, thrombin causes more platelet activation [31]. Congenital thrombocytopathies may disrupt the platelet hemostatic mechanisms, leading to bleeding [32] or, in rare cases, to thrombosis. For instance, sticky platelet syndrome is characterized by hyperaggregability that leads to arterial and venous thrombotic events [33].

### 2.1. Factor V Leiden

Factor V (FV) plays an important role in the coagulation process, acting as a cofactor for factor Xa in the prothrombinase complex and leading to thrombin generation. A single nucleotide polymorphism (1691G>A) in the FV gene determines the synthesis of an abnormal protein: the FV Leiden. The procoagulant activity of FV Leiden is not affected, but the arginine-to-glutamine substitution occurs at an important protein’s cleavage site and significantly reduces the rate at which FVa is deactivated by activated protein C (APC) [34,35]. Factor V Leiden also decreases the APC cofactor activity of FV in the deactivation of factor VIIIa. The association between impaired FVa and VIIIa deactivation results in a prothrombotic state [36].

Factor V Leiden represents the most frequent cause of inherited thrombophilia. The prevalence is 3–5% in the general population, 20% in patients with deep venous thrombosis and about 50% in patients with familial thrombophilia [37,38]. In the general population, the prevalence of FV Leiden varies considerably with geographic area, ethnic group, and the degree of ethnic mixture within ethnic groups [39,40,41]. The highest prevalence is found in Caucasians and the lowest in Asians.

An association between ONFH and the genetic polymorphisms in FV has also been reported. One prospective cohort study assessed 244 USA patients with ONFH (unilateral or bilateral disease) and found that 9.3% of 161 patients with idiopathic ONFH and 9.6% of 83 patients with secondary ONFH (known trauma, alcoholism or long-term and/or high-dose corticosteroids) had FV Leiden, thereby supporting the hypothesis that FV Leiden is a risk factor for ONFH [42].

Another study evaluated 68 adult patients with ONFH, of which 63 had nontraumatic etiologies. Out of 33 patients with secondary ONFH (known trauma, alcoholism or corticosteroids use), only one patient had FV Leiden. Meanwhile, out of 35 patients with idiopathic ONFH, 8 patients had FV Leiden, including one patient who also had the prothrombin 20210A gene mutation [43]. In all cases, the patients were heterozygous for the gene mutations. In patients with idiopathic ONFH, FV Leiden was significantly more prevalent than in patients with corticosteroid-induced or alcohol-induced ONFH [43]—and doubly as prevalent as in the control group, where 32 cases of FV Leiden were identified among 282 healthy volunteers [26]. This increased prevalence can be explained by the characteristics of the population enrolled in the study, which was from southern Sweden—a region where the prevalence of FV Leiden is 10%, one of the highest rates in the world.

In a study that enrolled 72 consecutive Caucasian Greek patients with nontraumatic ONFH and 300 healthy subjects [44], FV Leiden was present in 18.0% of ONFH patients (all heterozygous) and in 4.6% in the control group (4.3% heterozygous, 0.3% homozygous), with OR 4.5 (95% CI: 2.0–10.0), thus directly linking the procoagulant status determined by FV Leiden to ONFH. The prevalence of FV Leiden was significantly increased both in primary and secondary ONFH patients, with the highest (21.7%) in the idiopathic ONFH subgroup, who showed a significant OR of 5.7 (95% CI: 1.8–17.5).

A European study of unrelated adult Caucasians of Polish origin included 45 patients with idiopathic ONFH and 23 patients with secondary ONFH, and reported no association between FV Leiden and ONFH [45].

Major thrombophilic mutations like FV Leiden have been identified as risk factors for nontraumatic ONFH in Caucasians, but have not been confirmed in Asian populations. Not only is prevalence of FV Leiden very low in the general Asian population [39], but studies in Asian populations did not confirm any association between FV Leiden and ONFH. In the Korean general population, the mutation has not been identified—nor has it been identified in ONFH patients or control subjects [46]. This finding supports the results of a previous study, in which 418 Koreans (including normal individuals, with thrombotic diseases and with nonthrombotic disorders) were screened for the presence of FV Leiden. The mutation was not identified in any case [47]. Similar results were provided by a Chinese study that included 267 patients with venous thromboembolism (VTE) and 102 control subjects. Although FV Leiden is a known risk factor for VTE, none of the Chinese patients enrolled was a carrier of FV Leiden [48]. Therefore, the presence of FV Leiden can be considered a risk factor for ONFH primarily in Caucasians. Its existence should be sought in patients with idiopathic ONFH within this ethnic group.

### 2.2. Prothrombin G20210A Mutation

The presence of the G20210A prothrombin gene mutation is responsible for high levels of prothrombin, which leads to an increased generation of thrombin and to a prothrombotic state. This mutation is present in 1–5% of Caucasians and is associated with deep venous thrombosis [49] and pulmonary embolism [50].

A prospective North American cohort study that evaluated the presence of prothrombin G20210A mutation in 235 patients with idiopathic and secondary ONFH found that 8 (3.4%) were heterozygous for prothrombin G20210A. This was similar to the control group, where 3 of 104 subjects (2.9%) had the mutation [42]. Therefore, in this study, the prothrombin G20210A mutation alone was not a significant risk factor for ONFH, which confirmed the same author’s previous results [51].

A North European study evaluated 68 adult patients with idiopathic and secondary ONFH (known trauma, alcoholism or corticosteroid use). Only four patients had the prothrombin G20210A mutation (three with idiopathic ONFH and one with posttraumatic ONFH) [43]. Therefore, an independent association of prothrombin G20210A mutation with ONFH could not be established. However, when analyzed together, the presence of FV Leiden and prothrombin G20210A mutation in patients with idiopathic ONFH showed a clear pattern. These two major thrombophilic mutations occurred with more than twice the frequency of the control subjects [26,52] and were 10 times more common than in patients with corticosteroid-induced or alcohol-induced ONFH (OR 10.8; 95% CI, 1.4–84) [43], suggesting that the presence of a thrombophilic substrate is a favoring factor for ONFH.

In a study that enrolled 72 consecutive Caucasian Greek patients with nontraumatic ONFH and 300 healthy subjects [44], prothrombin G20210A mutations were identified in 4.2% of ONFH patients (1.4% heterozygous, 2.8% homozygous) and in 2.6% in the control group (2.3% heterozygous, 0.3% homozygous), with OR 1.6 (95% CI 0.4–6.1). Although statistical significance was not achieved and no significant association between prothrombin G20210A mutation and ONFH could be established, a considerably higher prevalence of prothrombin G20210A mutation (8.7%) was found in patients with idiopathic ONFH [44]. A European study of unrelated adult Caucasians of Polish origin included 45 patients with idiopathic ONFH and 23 patients with secondary ONFH. It also reported no association between prothrombin G20210A mutation and ONFH [45].

The mutation has not been identified in Korean populations, ONFH patients, or control subjects [46]. Similar, a Chinese study that included 267 patients with VTE and 102 control subjects showed that none of the patients enrolled was a carrier of the prothrombin G20210A mutation [48].

Important conclusions can be drawn from the available studies; namely, that the prevalence of the G20210A mutation in the prothrombin gene in patients with ONFH varies between ethnic groups and that it alone is not a risk factor for ONFH. However, it exerts a thrombogenic effect when added to other thrombophilic mutations.

### 2.3. Antithrombin III Deficiency

Antithrombin III (ATIII) is a circulating anticoagulant glycoprotein synthetized in the liver and secreted in plasma [53]. The main action of ATIII is the inhibition of thrombin and factor Xa. It also has minor inhibitory effects on factors IXa, XIa, and XIIa. The two ATIII active sites are the reactive site, responsible for its proteolytic function, and the heparin binding site. In the presence of heparin, the ATIII anticoagulant activity is increased more than 1000-fold [54], thus enhancing the inactivation of thrombin and factor Xa.

The ATIII deficiency can be inherited or acquired. The inherited form is an autosomal dominant condition caused by a defected allele of the *SERPIN1* gene, and is classified into two types. Type 1 is defined by the absence of gene product in a homozygous state, while in the heterozygous case, normal antithrombin activity is approximately halved. Type 2 is characterized by a qualitative inadequate protein. Various conditions may lead to acquired ATIII deficiency, including liver disease, malnutrition, nephrotic syndrome, sepsis and treatment with L-asparaginase [54,55].

ATIII deficiency leads to a hypercoagulable state that significantly increases thrombotic risk [56]. In fact, ATIII deficiency has been associated with a 16.3-fold increase in the VTE risk) compared to nonthrombophilic individuals) [57].

The association between ATIII deficiency and ONFH was also considered in different clinical settings [58,59,60,61]. Chotanaphuti et al. identified two cases of ATIII deficiency out of 40 Thai patients with idiopathic ONFH [60]. In Canadian patients with nontraumatic ONFH, Séguin et al. found that the ATIII deficiency was present in only one patient out of 49 included [62]. It is of note that, in their study, all hypercoagulability markers had low prevalence. The study of Garcia et al. enrolled 24 Brazilian patients with ONFH and identified zero cases with ATIII deficiency [63]. Very recent published data by Rathod et al. [59] showed a lower prevalence of ATIII deficiency in idiopathic ONFH patients compared to healthy controls (11% vs. 22%), suggesting that, when familial thrombophilia is considered a substrate of primary ONFH, the involvement of ATIII deficiency is unlikely.

Although the incidence of ATIII deficiency has been intensively investigated in idiopathic and secondary ONFH, studies have proved the scarcity of ATIII deficiency among ONFH patients, implying that its contribution is a minor one in the development and progression of the disease.

### 2.4. Protein C and Protein S Deficiency and Resistance to Activated Protein C

Protein C is a glycoprotein synthesized by hepatocytes through a vitamin K-dependent pathway and circulates in blood as zymogen [64]. It is activated by thrombin bound to thrombomodulin, which is a catalytic cofactor present on endothelial cells’ surface membranes [65]. APC exerts its anticoagulant effect by degrading factors Va (FVa) and VIIIa (FVIIIa) on the surface of negatively charged phospholipid membranes using lipid and protein cofactors. Protein S, also a vitamin K-dependent plasma protein, is synthesized by the liver, endothelial cells and megakaryocytes. It is a cofactor for APC, enhancing the inactivation of factors Va and VIIIa on phospholipid surfaces.

While for FVa inactivation, only protein S is an important APC cofactor, inactivation of FVIIIa is more complex, and requires both protein S and the intact FV molecule as synergistic APC cofactors [66]. Therefore, mutations in FV genes can disrupt the functionality of the C protein pathway. The Gln506 mutation of the FV gene is common and results in FV Leiden, while Arg306 mutation of the FV gene is less common and results in resistance to activated protein C [67]. It can be considered that FV Leiden and resistance to activated protein C represent the same type of thrombophilia, as they are the consequences of different mutations of the FV gene. While FV Leiden is commonly associated with the pathogenesis of ONFH, there is no data showing a link between acquired activated protein C resistance and the risk of ONFH—although it has been described as a risk factor for thrombosis [60,68,69,70].

Protein C and protein S deficiency, as well as activated protein C resistance, through the hypercoagulability status that they induce, may underlie the pathogenic mechanism that leads to bone necrosis in patients with ONFH [60,68,69,71].

The first case of protein S deficiency associated with ONFH in adults was reported in 1997 by Pierre-Jacques et al. [72]. In the same year, Glueck et al. reported that protein S deficiency was found in 7% of the adults with ONFH from the study group, while protein C deficiency was identified in 2–13% of cases [68]. Zalavras et al. found that patients with idiopathic ONFH had decreased protein C and S levels compared to control group. Protein C deficiency was present in 29.4% of cases with primary ONFH and in 21.6% of cases with secondary ONFH. Protein S deficiency was present in 5.9% of cases with primary ONFH and in 11.8% of cases with secondary ONFH [73]. Similar, Garcia et al. found that patients with idiopathic ONFH were 5 times more likely to have protein S deficiency and 2.14 times more likely to have protein C deficiency than patients with secondary ONFH [63].

In 106 patients with ONFH, Korompilias et al. identified 88 cases with coagulation abnormalities: 35 patients (33%) showed resistance to activated protein C alone and 2 patients (1.9%) had protein S deficiency [74]. Rathod et al. also confirmed the association between protein C or S deficiency and ONFH, with statistically significant differences between ONFH patients and the control group (*p* value = 0.028 for protein C deficiency and *p* value = 0.038 for protein S deficiency) [59].

All the available and relevant data demonstrate a strong association between protein C and protein S deficiency—as well as activated protein C resistance and ONFH. Studies have shown that the most common familial thrombophilia associated with osteonecrosis are the FV Leiden mutation and/or RAPC. These were found in 15.5% of cases in a series of 535 patients with ONFH [75].

### 2.5. MTHFR C677T Gene Polymorphism

The enzyme 5,10-methylenetetrahydrofolate reductase (MTHFR) is vital to the homocysteine metabolism pathway. Gene mutations can reduce the activity of the enzyme and cause the accumulation of homocysteine in the blood, which leads to an increased production of peroxides and oxygen free radicals via self-oxidation of homocysteine. Reactive oxygen species injure endothelial cells and disrupt the balance between vasodilator and vasoconstrictor endothelium-derived factors by increasing endothelin-1 secretion and decreasing relaxing factor and prostaglandin secretion. This imbalance further triggers thrombosis [76]. Moreover, the thrombomodulin’s action on the endothelial surface is reduced, leading to a decrease in protein C activity and the inhibition of factors Va and VIIIa. The thrombin generation is thus intensified, resulting in increased fibrin formation [77]. Conversion of fibrinogen to insoluble polymer fibrin gives structural stability, strength, and adhesive surfaces for growing blood clots [78].

Since MTHFR C677T gene polymorphism leading to hyperhomocysteinemia has been associated with deep venous thrombosis and pulmonary embolism [79,80,81], special attention has been paid to its potential influence on ONFH occurrence. While some studies have shown a correlation between MTHFR C677T gene polymorphism and ONHF [44,45,46,82], others have had neutral [51] or opposite results [83,84].

A meta-analysis that included eight studies evaluating the relation between MTHFR C677T gene polymorphism and ONFH (778 cases and 1162 controls) reported the absence of a significant association when all subjects were analyzed, but highlighted the presence of an ethnicity-dependent risk. There was an association between MTHFR C677T gene polymorphism and ONFH risk in non-Asian populations (OR = 1.72; 95% CI: 1.21–2.43), but not in Asian populations (OR = 0.88; 95% CI: 0.66–1.66) [85].

The most recent meta-analysis that investigated the correlation between MTHFR C677T gene polymorphism and nontraumatic ONFH analyzed the results of 11 studies and confirmed the association between this genetic substrate and the risk of ONFH. The OR was 0.72; 95% CI: 0.54–0.96 for the entire population included. In an analysis of different ethnic groups, MTHFR C677T gene polymorphism was associated with ONFH, especially in Caucasian subjects. Caucasians with the CC genotype had a lower risk for ONFH than subjects with CT+TT genotype, with an OR = 0.65 [86].

These results can be explained by the variable prevalence of the mutation with ethnicity. The prevalence is high among Caucasians (32.2–44%) and Asians (30.5–42%) and low in Africans (6–10.3%) [87,88]. Moreover, the level of homocysteine in the blood is influenced by a series of factors, such as drugs, smoking, alcohol consumption and diet. Because MTHFR participates in the homocysteine metabolism alongside folates and vitamin B12 [89], persistent low levels of folates and B12 may lead to hyperhomocysteinemia and increased thrombotic risk. Therefore, regional eating habits (resulting in diets deficient in these compounds) and alcoholism may be associated with increased homocysteine levels and can influence the prevalence of ONFH related to MTHFR gene polymorphism.

In the presence of many confounding factors, it is very difficult to separate and accurately analyze the contribution of the genetic substrate, independent of environmental factors. Therefore, the magnitude of its impact on the onset of ONFH cannot be accurately assessed—although we know that the MTHFR C677T gene polymorphism has thrombogenic potential.

## 3. Hypofibrinolytic Disorders

Thrombus formation and dissolution is a dynamic and continuous process in the human body. While activation of the coagulation cascade leads to formation of the fibrin clot, the activation of the tissue plasminogen activator (t-PA) on plasminogen-binding sites initiates fibrinolysis. The release of t-PA from endothelial cells leads to the conversion of proenzyme plasminogen into plasmin [90]. Fibrinolysis is a highly regulated process. The role of plasminogen activator inhibitor-1 (PAI-1) is to control plasmin activity by inhibiting t-PA. If the PAI-1 level is increased, the t-PA activity and clot degradation decrease [91]. The hypofibrinolysis state (determined by high levels of PAI-1) leads to increased clot formation and thrombotic venous outflow obstruction in the bone.

Main hypofibrinolytic disorders include high levels of PAI-1 and lipoprotein(a). Due to its structural similarity to plasminogen, lipoprotein(a) may exert an inhibitory effect on fibrinolysis, thus facilitating thrombosis. While studies focusing on the relation between lipoprotein(a) and the risk of VTE have shown contradictory results [92,93,94,95], the association between high levels of PAI-1 and the risk of VTE has amassed significant evidence [96,97].

### Plasminogen Activator Inhibitor-1

Plasminogen Activator Inhibitor-1 (PAI-1) is a protein synthesized and released by endothelial cells which functions as a serine protease inhibitor [98]. It is the primary inhibitor of circulating plasminogen activators, t-PA and urokinase (u-PA). PAI-1 exerts its effects mainly through the formation of complexes with t-PA, reducing the ability of t-PA to convert plasminogen to plasmin. Suppression of plasmin generation results in hypofibrinolysis and a relative state of hypercoagulability. As PAI-1 is an inhibitor of fibrinolysis, high levels of PAI-1 have been associated with both arterial and venous thrombosis [99,100,101]—and also with ONFH [14,51].

The PAI-1 gene has three genotypes: 4G4G, 4G5G, and 5G5G, the consequence of a single guanosine nucleotide insertion/deletion variation at bp 2675 of the PAI-1 promoter [102]. The 4G/5G polymorphism of the PAI-1 gene correlates with susceptibility to ONFH. As the 4G4G genotype is associated with higher plasma PAI-1 activity than the other two genotypes, the 4G4G carriers exhibit reduced plasma fibrinolytic activity and are more prone to thrombotic events [103].

The 4G/5G polymorphism of the PAI-1 gene has been linked to OHNF for some time. Patients with ONFH were more likely to have heterozygosity and homozygosity for the hypofibrinolytic 4G polymorphism of the PAI-1 gene than control subjects [82]. Glueck et al. reported in the late 1990s that 41% of 59 patients with ONFH were homozygous for the 4G/4G polymorphism (versus 20% of 40 healthy control subjects) and that 61% of patients had high PAI-1 activity (versus 5% of control subjects) [51].

Since then, more studies have confirmed that PAI-1 gene 4G/5G polymorphism is strongly correlated with the risk of ONFH. The meta-analysis of Liang et al. included five studies (419 cases and 969 controls) that systematically evaluated the association between 4G/5G polymorphism in the PAI-1 gene and the risk of ONFH. The analysis indicated increased risk of ONFH in the 4G4G genotype carriers, but not in 4G5G genotype carriers, as compared with 5G5G homozygotes [102]. While the subgroup analysis by ethnicity indicated no significant association between PAI-1 polymorphism and ONFH risk among Asians, a positive significant statistic association was found among Caucasians (*p* = 0.005 for comparisons 4G5G vs. 5G5G allele; *p* = 0.000 for comparisons 4G4G + 4G5G vs. 5G5G allele). This suggests that the substrate of the hypercoagulability state in ONFH patients could be subjected to ethnically-driven genetic differences and that Caucasians’ 4G4G carriers have an increased risk of ONFH.

Further studies showed that the risk of ONFH in subjects with the 4G allele was 1.76-fold higher than that of subjects with the 5G allele [104].

A very recent meta-analysis by Sobhan et al. [105] strongly reinforced previous results. A total of six studies with 456 cases and 1019 controls were analyzed, comprising both Caucasian and East Asian subjects (three studies from each ethnic group). A significant association between PAI-1 4G/5G polymorphism and ONFH risk was found, in overall population and by ethnicity.

Most of the evidence accumulated so far confirms the association between the PAI-1 4G/5G polymorphism and the risk of ONFH [14,51,104,105,106]. On the contrary, a study conducted in a Japanese population [83] found no association, emphasizing the importance of genetic backgrounds among the pathological conditions that determine ONFH.

## 4. Therapeutic Implications

The association between hereditary thrombophilia-hypofibrinolysis and ONFH was confirmed by robust evidence accumulated over time from clinical trials and isolated case reports, allowing the expansion of therapeutic options by adding anticoagulant treatment. As anticoagulation has been shown to be effective in the prevention and treatment of VTE (with or without associated hereditary thrombophilic conditions), a similar approach has been used in patients with osteonecrosis. The working hypothesis is that anticoagulant treatment may limit the progression of intraosseous venous thrombosis, allowing the spontaneous lysis of thrombi. That, in turn, improves blood circulation and bone nutrition.

To provide up-to-date information on the use of anticoagulant therapy in patients with ONFH, we performed a search in the Web of Science database to retrieve relevant studies regarding anticoagulant treatment in patients with ONFH and hereditary thrombophilia-hypofibrinolysis. The keywords “osteonecrosis” and “anticoagulant” or “anticoagulation” were automatically searched in the titles and abstracts of the articles. The titles and abstracts of all 83 search results were further manually screened, removing duplicate information, articles focusing on osteonecrosis with locations other than the femoral head, review articles and meta-analyses. In the final analysis, we included eight original papers reporting results of anticoagulant use in patients with ONFH and hereditary thrombophilia-hypofibrinolysis (Table 1). The reference lists of the included publications were also screened for additional potential suitable publications.

The first published studies and case reports on this subject were in the late 1990s. Several pilot studies by Glueck et al. using warfarin and stanozolol (a synthetic derivative of testosterone) in the treatment of osteonecrosis of the hip and jaw showed that therapeutic benefits (i.e., pain relief) occurred in the first 12 weeks of treatment [3,107]. In 26 patients with osteonecrosis of the mandible and maxilla, the chronic disabling facial pain was ameliorated in 60% of patients with hereditary thrombophilia treated with the oral vitamin K antagonist warfarin and in 60% of patients with hypofibrinolysis treated with stanozolol [113]. The authors pointed out that if no clinical benefits were achieved after 12 weeks, continuing treatment for up to 16–24 weeks was not justified because no additional benefits were added. Furthermore, in patients with ONFH and bone collapse (Ficat stages III or IV) the medical therapy was not associated with any benefit, and therefore should not be recommended in patients with irreversible bone damage [3,107].

Studies published later (by the same authors) regarding the use of enoxaparin in patients with osteonecrosis highlighted the benefits of anticoagulant treatment in alleviating pain and reducing the progression of osteonecrosis. 24 patients with ONFH caused by heritable thrombophilia or hypofibrinolysis (Ficat stages I or II) received enoxaparin 60 mg od. for 12 weeks. The choice of enoxaparin as an anticoagulant and the dose were determined by data available at that time on its use in the prophylaxis of VTE in patients undergoing hip surgery [114]. The 12-week treatment duration overlapped with the minimum period of anticoagulant treatment in VTE. The enoxaparin treatment reduced pain in 82% of hips and imaging evaluations confirmed the unchanged aspect of the femoral head in 76% of hips at a 36-week follow-up. At the end of the follow-up period, 82% of hips had not been affected by segmental femoral head collapse. The study showed the efficacy of enoxaparin without any safety concerns [107].

An unblinded pilot study with enoxaparin [25] enrolled 28 patients with ONFH, Ficat stages I or II, and thrombophilic or hypofibrinolytic disorder. Of these, 16 patients (25 hips) had primary osteonecrosis and 12 patients (15 hips) had osteonecrosis secondary to corticosteroid use. The intended follow-up period was ≥108 weeks, but this goal could not be achieved in 12 patients (3 with primary and 9 with secondary ONFH). Very good results were achieved in patients with primary ONFH, in which 95% of hips had a stationary evolution at ≥180-week follow-up. This demonstrated the existence of a major benefit of anticoagulation, as compared to the natural history of the disease, in which, at 2 years, only 20–50% of hips are typically preserved [25,115]. In patients with secondary ONFH, the natural evolution of the disease was unchanged, with 80% of hips progressing to Ficat stages III or IV.

When 20 patients (30 hips) with primary osteonecrosis, Ficat stages I or II, and thrombophilia-hypofibrinolysis state were followed 4–7 years after receiving enoxaparin, the results were even more encouraging. For 3 months, 16 patients (25 hips) received enoxaparin 60 mg od. and 4 patients (5 hips) received enoxaparin 1.5 mg/kg daily. Based on intent to treat, 80% of hips remained unchanged at a 4-year follow-up, and 60% of hips remained unchanged at a 7-year follow-up [109], confirming the hypothesis that 3 months of anticoagulant therapy leads to long-term benefits.

This hypothesis was also supported by data from the very long-term follow-up (4–16 years) of 6 patients (9 hips) with hereditary thrombophilia and continuous anticoagulant therapy warranted by unprovoked recurrent thrombotic events (≥ 2 prior thrombotic events). Five patients were heterozygous for FV Leiden and 1 had RAPC. All patients had Ficat stages I–II idiopathic ONFH. The patients received 3 months of enoxaparin, followed by long-term oral anticoagulation. A vitamin K antagonist (with the INR targeted between 2 and 3) was used in 5 cases, one of which was switched to to rivaroxaban 20 mg od. after three years. One patient received 150 mg bid of dabigatran etexilate [75]. At the follow-ups (4, 4, 9, 13, 13, and 16 years, respectively, for the 6 cases included in the long-term anticoagulation study), the Ficat stage remained unchanged. No hip collapses and no progressions to osteoarthritis were objectified at successive imaging evaluations (X-rays and MRIs). The patients were symptom-free after 3–16 months of anticoagulant treatment, and exhibited full active range of motion (except for one patient with persistent pain when active).

The efficiency of direct oral anticoagulants was highlighted in other case reports, as well. One patient with important thrombophilic burden and history of deep venous thrombosis (anticoagulated with warfarin for atrial fibrillation and symptomatic for ONFH) showed complete resolution of symptoms after 8 months on apixaban 5 mg bid [111]. Milgrom et al. [110] reported the case of a woman diagnosed at the age of 26 with primary multifocal ON, in context of complex thrombophilia (FV Leiden heterozygosity, C677T MTHFR homozygosity, and hypofibrinolytic 4G4G homozygosity for the PAI-1 gene). After 3 months of enoxaparin 40 mg bid, the anticoagulant treatment was continued with dabigatran etexilate 150 mg bid. The pain improved notably after 10 months of oral anticoagulation and, at a 6-year follow-up, the patient was free of joint pain, with good functional mobility, and without osteonecrosis progression at the imaging evaluation.

More favorable results on long-term use of anticoagulant therapy in ONFH patients with hereditary thrombophilia were very recently published [112]. Nine patients (13 hips) with Ficat stages I or II and primary ONFH (8 patients with FV Leiden heterozygosity and 1 with prothrombin G202010A heterozygosity) received enoxaparin 60 mg od. for 3 months. Four patients continued the anticoagulation with warfarin (INR 2–2.5), three with direct oral anticoagulant and one with enoxaparin 120 mg od. After a follow-up period of 12 ± 5 years, no hip progressed to collapse and in one hip the X-ray aspect normalized. Patients became symptom-free after 3–10 months of anticoagulant treatment and they remained so throughout the follow-up period.

Other studies also support the favorable results of anticoagulant therapy in terms of improving symptoms and functional status, and limiting the progression of bone damage. 36 patients with primary ONFH Ficat stages I or II were included in a comparative retrospective study [108]. 18 patients (26 hips) were treated with 60 mg od. enoxaparin for 3 months and 18 nonanticoagulated patients (23 hips) were followed-up for a minimum of 24 months. In the enoxaparin group, 15 hips (57.7%) remained in the pre-collapse stage, while in the control group, only 5 hips did so (21.7%), *p* = 0.042. Although a thrombophilic substrate was only objectified in 38.9% patients from the enoxaparin group and 27.8% of the control group, this study offered more evidence on the benefits of anticoagulation in reducing ONFH progression.

A case report of a 40-year-old Caucasian woman with osteonecrosis in the distal femur and homozygosity for the 4G variant in the PAI-1 gene was published by Haydock et al. [116]. The patient received full anticoagulant treatment with enoxaparin, 1 mg/kg bid for 6 months, and experienced major relief of symptoms. As the bone pain reappeared a few months after stopping treatment, anticoagulation was resumed with a prophylactic dose of apixaban 2.5 mg bid. The outcome was good; the patient was free of bone pain and showed no osteonecrosis progression at her 1-year follow-up.

Similar favorable results were obtained in 6 patients with stage II knee osteonecrosis and hereditary thrombophilia-hypofibrinolysis [117]. Enoxaparin treatment had favorable effects on pain (4 out of 6 patients were pain-free), prevented bone collapse and progression to severe osteoarthritis and led to restoration of full function. During a follow-up period of 2–15.1 years, four patients became and remained asymptomatic. One patient with no symptomatic benefit on enoxaparin was switched to direct oral anticoagulant and improved on rivaroxaban at 1-year follow-up.

## 5. Discussion

The goals of ONFH treatment are pain control and preservation of the femoral head. One study showed that, 2.27 years after diagnosis, 55.9% of all patients and 46.2% of patients with primary ONFH developed symptoms [118]—and this data was confirmed by the results of a meta-analysis evaluating 664 hips [119]. The progression to symptoms or to bone collapse occurred in 59% of hips [119]. The natural history of untreated ONFH is that 60% to 80% of patients who start at Ficat Stage II (no hip collapse) will rapidly progress to Ficat stage III or IV (segmental collapse of the femoral head/secondary arthritis) requiring total hip replacement within 2 years of initial diagnosis [25,115].

From the beginning, medical preservation of hips (by acting on pathogenic mechanisms) has been an essential goal. When the theory of intraosseous vascular thrombotic occlusion was developed [3,4], the anticoagulant treatment became a new therapeutic option. It was hypothesized that hereditary thrombophilia allowed thrombus formation in the bone veins. Hereditary thrombophilia is characterized by an increased tendency to form thrombi and hypofibrinolysis, in which the ability to lyse thrombi is reduced. The intraosseous venous pressure increases and reduces the arterial blood flow. Persistent osseous hypoxia and lack of nutrients determine bone ischemia that leads to bone necrosis and collapse of the articular surface [107].

The presence of a hereditary or acquired prothrombotic state significantly increases the risk of VTE. Antithrombin III, protein C and S deficiencies are associated with a 7–8-fold increase in the risk of venous thromboembolic events [120,121,122]. The presence of FV Leiden causes a 7-fold increase in the risk of deep venous thrombosis in heterozygotes [123] and an 80-fold increase in homozygotes [124].

Similar to VTE patients, the presence of hereditary thrombophilia (FV Leiden, G20210A prothrombin, low antithrombin III, protein C and S levels, resistance to activated protein C and high homocysteine level) has been correlated to ONFH. There are also data that suggest that the high PAI-1 levels that characterize the state of hypofibrinolysis leads to ONFH.

Nowadays, anticoagulant treatments are successfully used in VTE prevention and treatment [125,126,127]. In patients with homozygous FV Leiden or homozygous prothrombin G20210A mutation or with other hereditary thrombophilic states (such as deficiency of antithrombin III, protein C, or protein S), indefinite anticoagulant treatment is recommended after a first episode of pulmonary embolism occurring in the absence of a major reversible risk factor [125].

In patients with ONFH and hereditary thrombophilia-hypofibrinolysis, the role of anticoagulant treatment is to limit the progression of intraosseous venous thrombosis, allowing the spontaneous lysis of thrombi. It is hoped that this process will improve blood flow and bone ischemia. The bone will heal and recover, respecting the normal architecture [128]. The progress of osteonecrosis will slow or stop, avoiding total joint replacement.

As early studies showed, for the treatment to bring benefits, the anticoagulant treatment should be started in the early stages of osteonecrosis (Ficat stages I or II) [128], before irreversible bone changes occur. If segmental collapse of the head of the femur is present (Ficat stages III or IV), no benefit is obtained from the use of anticoagulant treatment [107]. Early intervention is even more important, as studies show that even asymptomatic osteonecrosis has a high rate of progression, regardless of its underlying cause (primary or secondary). Additionally, anticoagulant therapy has been shown to be particularly effective in patients with primary ONFH, but not in those with secondary ONFH, in whom the rate of progression to Ficat stages III or IV has been unchanged by anticoagulation [25].

Short term (three months) use of enoxaparin was associated with important improvements; patients were free of pain, and showed improved mobility or regained full mobility. The absence of disease progression was objectively confirmed by X-Ray and MRI exams [25,114]. Three months of anticoagulant therapy also provides long-term benefits (4–7 years) [75].

Recent data—albeit on a small number of patients—showed that continuous anticoagulation stopped the progression of osteonecrosis, reduced or removed pain, and allowed patients to regain full activity and range of motion. Additionally, symptoms were relieved and full function was regained in almost every case, the collapse of the head of the femur was prevented and the progression of idiopathic ONFH was stopped. The anticoagulant treatment preserved the joints from progressive osteonecrosis, thus reducing the need for total hip arthroplasty [112].

In terms of safety, no hemorrhagic events were reported during anticoagulant treatment, except for one case of hematuria (with spontaneous resolution) reported by Chotanaphuti et al. [108].

Over time, parallel to the evolution of anticoagulants, studies and case reports have demonstrated benefits in using warfarin, low-molecular-weight heparin (LMWH) and recently, direct oral anticoagulants (DOAC) in patients with ONFH [25,75,107,110,111,112]. There are two types of DOACs available on the market for VTE prophylaxis and treatment. Dabigatran is an inhibitor of factor IIa, while apixaban, edoxaban and rivaroxaban are inhibitors of factor Xa. Large randomized trials with these DOACs in patients with VTE have encouraged their use, as they are at least as effective as warfarin, but have a better safety profile, especially in reducing the risk of intracranial bleeding [129,130,131,132].

Long-term treatment with LMWH, although frequently studied and used, carries some drawbacks, such as the need for daily injection. The new generation of anticoagulants has many advantages: oral administration in fixed doses, stable effects, no need for routine monitoring, and few food/drug interactions. Case reports have already been published in which ONFH evolved favorably under different types of DOAC [75,110,111,116,117].

More and more patients with VTE are being treated with DOAC instead of vitamin K antagonists. As such, we will probably assist in coming years with this growing shift in the anticoagulant treatment of patients with ONFH. Enoxaparin will likely be replaced by a DOAC and, given the ease of administration, efficacy and safety, the number of patients with ONFH who are treated with anticoagulants is expected to increase as well.

The results of numerous studies have shown that anticoagulant treatment in primary ONFH initiated before irreversible bone collapse may change the natural history of the disease in patients with hereditary thrombophilia-hypofibrinolysis. Therefore, not only should the presence of a thrombophilic substrate be sought in patients with primary ONFH but, if identified, the assessment should be extended to grade I family members. The search for thrombophilic substrates should be performed in parallel with the imaging evaluation of the hips, because offspring with thrombophilic gene mutations carry an increased risk of osteonecrosis of the femoral head. Early diagnosis and therapeutic intervention prevents or delays the progression of the disease, improving the quality of life and reducing the need for arthroplasty.

## 6. Conclusions

The definite association between ONFH and hereditary thrombophilia-hypofibrinolysis—as well as increased understanding of the mechanism that leads to venous thrombosis, bone ischemia, necrosis and collapse of the femoral head—has made possible the use of anticoagulant therapy in patients. Undeniable benefits have been obtained, e.g., no pain, recovery of joint mobility and slowing/stopping of the progression of the disease the pre-collapse stage. Therefore, it is important that patients with primary ONFH in the pre-collapse stage be screened for hereditary thrombophilia-hypofibrinolysis. In this way, they can benefit from early anticoagulant therapy if the thrombophilic substrate is confirmed. Thrombophilia and ONFH screening should also be considered in first-degree relatives of already-diagnosed patients in order to allow for early evaluation and therapeutic intervention.

## Figures and Tables

**Table 1:** Methods and outcomes of studies on the use of anticoagulants in patients with ONFH.

Author, Year	No. Patients	Disease Severity	Etiology of Osteonecrosis	Anticoagulant	Study Duration	Efficacy	Safety
Glueck et al. 2001 [107]	24 patients	Ficat stages I–II	Heritable thrombophilia and/or hypofibrinolysis	Enoxaparin 60 mg od. 12 weeks	36 weeks	76% of hips were unchanged and 24% of hips were worse at 36-week follow-up 82% of hips were without femoral head collapse at 36-week follow-up Enoxaparin reduced pain in 82% of hips	No enoxaparin-related side effects
Glueck et al. 2005 [25]	28 patients (40 hips)	Ficat stages I–II	16 patients (25 hips)—primary osteonecrosis: familial or acquired thrombophilic or hypofibrinolytic disorder or both 12 patients (15 hips)—secondary osteonecrosis (long-term high-dose corticosteroid use)	Enoxaparin 60 mg od. 12 weeks	≥180 weeks	95% of hips were unchanged at ≥180-week follow-up in primary ONFH (76% based on intent to treat) 80% of hips worsened at ≥180- week follow-up in secondary ONFH	No bleeding episodes, anemia or thrombocytopenia
Chotanaphuti et al. 2013 [108]	18 anticoagulated patients (26 hips) and 18 non-anticoagulated patients (23 hips)	Ficat stages I–II	Thrombophilic substrate in 38.9% of patients from the enoxaparin group and in 27.8% of patients from the control group	Enoxaparin 60 mg od. 3 months	24 months	15 hips (57.7%) remained in the pre-collapse stage in the enoxaparin group 5 hips (21.7%) remained in the pre-collapse stage in control group (*p* = 0.042).	Transient hematuria in one patient from the enoxaparin group
Glueck et al. 2014 [109]	20 patients (30 hips)	Ficat stages I–II	Heritable thrombophilia and/or hypofibrinolysis	Enoxaparin 60 mg od. —16 patients (25 hips) 1.5 mg/kg daily —4 patients (5 hips) 12 weeks	4–7 years	80% of hips were unchanged at 4-year follow-up (based on intent to treat) 60% of hips were unchanged at 7-year follow-up (based on intent to treat)	No significant bleeding episodes
Glueck et al. 2015 [75]	6 patients (9 hips)	Ficat stages I–II	5 patients—Factor V Leiden heterozygosity 1 patient—resistance to activated protein C	Enoxaparin 60 mg od. —5 patients 1.5 mg/kg daily —1 patient 3 months Followed by oral anticoagulation: VKA (INR 2–3) or rivaroxaban 20 mg od. or dabigatran etexilate 150 mg bid.	4–16 years (4,4,9,13,13,16 years respectively)	No Ficat staging progression No restriction in activities Full range of motion 5/6 cases were symptom-free after 3–16 months of anticoagulant treatment; the 6th patient required pain medication.	No significant bleeding episodes
Milgrom et al. 2017 [110]	1 patients (1 hip)	Ficat stages I–II	Factor V Leiden heterozygosity, C677T MTHFR homozygosity and hypofibrinolytic 4G4G homozygosity for the PAI-1 gene	Enoxaparin 40 mg bid. 3 months Followed by dabigatran etexilate 150 mg bid.	≥6 years	Pain improved after 10 months of oral anticoagulation No joint pain, good functional mobility, no osteonecrosis progression at the imaging evaluation at 6-year follow-up	No bleeding reported
Jarman et al. 2017 [111]	1 patient (2 hips)	Ficat stage I	Factor V Leiden heterozygosity, 4G/4G homozygosity for the PAI-1 gene, high ACLA IgM antibodies, eNOS T786C homozygosity	Warfarin 1 year than apixaban 5 mg bid.	8 months	Asymptomatic	No bleeding episodes
Glueck et al. 2020 [112]	9 patients (13 hips)	Ficat stages I–II	8 patients—Factor V Leiden heterozygosity 1 patientprothrombin G202010A heterozygosity	Enoxaparin 60 mg od. 3 months Folowed by warfarin (INR 2–2.5) —4 patients direct oral anticoagulant—3 patients enoxaparin 120 mg daily—1 patient	≥3 years (5–21 years)	No hip progressed to collapse Normalized X-ray aspect in one hip Symptom-free after 3–10 months of anticoagulant treatment	No bleeding reported

ONFH = osteonecrosis of the femoral head; MTHFR = methylenetetrahydrofolate reductase; PAI-1 = plasminogen activator inhibitor-1; ACLA = anti-cardiolipin antibody; eNOS = endothelial nitric oxide synthase.
